# Surgery and risk for multiple sclerosis: a systematic review and meta-analysis of case–control studies

**DOI:** 10.1186/1471-2377-13-41

**Published:** 2013-05-06

**Authors:** Carole Lunny, Jennifer A Knopp-Sihota, Shawn N Fraser

**Affiliations:** 1 Centre for Nursing and Health Studies, Faculty of Health Disciplines, Athabasca University, 1 University Drive Athabasca, Alberta, T9S 3A3, Canada

**Keywords:** Adenoidectomy, Appendectomy, Multiple sclerosis, Observational studies, Risk, Surgery, Systematic review, Tonsillectomy

## Abstract

**Background:**

Although the precise etiology of multiple sclerosis is largely unknown, there is some speculation that a prior history of surgery may be associated with the subsequent risk for developing the disease. Therefore, we aimed to examine surgery as a risk factor for the diagnosis of multiple sclerosis.

**Methods:**

We searched for observational studies that evaluated the risk for developing multiple sclerosis after surgery that occurred in childhood (≤ 20 years of age) or “premorbid” (> 20 years of age). We specifically included surgeries classified as: tonsillectomy, appendectomy, adenoidectomy, or “surgery”. We performed a systematic review and meta-analyses and calculated odds ratios (OR) and their 95% confidence intervals (CIs) using a random effects model.

**Results:**

We identified 33 case–control studies, involving 27,373 multiple sclerosis cases and 211,756 controls. There was a statistically significant association between tonsillectomy (OR = 1.32, 95% CI 1.08-1.61; 12 studies, I^2^ = 44%) and appendectomy (OR = 1.16, 95% CI 1.01-1.34; 7 studies, I^2^ = 0%) in individual’s ≤ 20 years of age and the subsequent risk for developing multiple sclerosis. There was no statistically significant association between risk for multiple sclerosis and tonsillectomy occurring after age 20 (OR = 1.20, 95% CI 0.94-1.53; 9 studies, I^2^ = 32%), in those with appendectomy at > 20 years (OR = 1.26, 95% CI 0.92-1.72; 5 studies, I^2^ = 46%), and in those with adenoidectomy at ≤ 20 years of age (OR = 1.06, 95% CI 0.68-1.68; 3 studies, I^2^ = 35%). The combined OR of 15 studies (N = 2,380) looking at “surgery” before multiple sclerosis diagnosis was not statistically significant (OR = 1.19, 95% CI 0.83-1.70; I^2^ = 71%).

**Conclusions:**

We found a small but statistically significant and clinically important increased risk for developing multiple sclerosis, in those with tonsillectomy and appendectomy at ≤ 20 years of age. There was no convincing evidence to support the association of other surgeries and the risk for multiple sclerosis. Well-designed prospective etiological studies, pertaining to the risk for developing multiple sclerosis, ought to be conducted and should include the examination of various surgeries as risk factors.

## Background

Multiple sclerosis (MS) is a complex immune-mediated inflammatory disease of the central nervous system affecting an estimated 2 million people worldwide [1]. MS, one of the most common non-traumatic neurological disorders in younger adults, affects more females (than males) with a population prevalence of approximately 0.1% in North America [1]. The epidemiological and clinical importance of MS lies in the significant disability and morbidity attributed to the disease. Not only is the condition relatively common, it is costly with an estimated economic burden exceeding $6 billion annually in the United States alone [2].

### Description of the condition

The first symptoms or signs of MS, diagnosed by a physician, are referred to as the clinical onset of the disease. As there are limited (and consistent) clinical, laboratory, and imaging findings in MS, diagnostic classifications are generally reduced to “definite MS” and “possible MS”. Several established diagnostic standards have been used over the years to assist physicians in the diagnosis of MS; these include criterion such as the Poser criteria [3] and most recently the McDonald criteria [4].

Although the precise etiology of MS is largely unknown, epidemiological studies point at an important role in both genetic and environmental factors that seem to act synergistically increasing an individual’s risk of developing the disease. Specifically, risks such as genetic factors (the presence of HLA-DRB1*15 alleles), infectious causes including Epstein-Barr virus (EBV), vitamin D insufficiency, exposure to cigarette smoke, and a more northern geographic residence (latitude) have all been well documented in the literature [5-9]. There is some speculation that other factors, including a history of tonsillectomy, appendectomy, or other surgery may also be associated with the risk of developing MS [10].

### Relevance of systematic review and meta-analysis

Although there have been studies examining prior surgery as a risk factor for MS, these studies have generally been smaller-scale observational studies of varied methodological quality in which a wide range of both positive and negative results have been reported. Therefore, we conducted a formal Cochrane style systematic review and meta-analysis of observational studies to examine the association between tonsillectomy, appendectomy, adenoidectomy, and other “surgery” and the risk of developing MS. We are not aware of another similar published meta-analysis on this topic; therefore, a systematic review (SR) including meta-analysis is timely.

## Methods

We followed the procedures for conducting systematic reviews and meta-analysis as outlined by the Cochrane Collaboration [11] and the reporting guidelines of the Meta-Analysis of Observational Studies in Epidemiology (MOOSE) [12].

### Search strategy

Studies were identified by several methods. First, we searched for completed reviews in the Database of Abstracts of Reviews of Effects (DARE), Evidence for Policy and Practice Information (EPPI) Centre, the HealthEvidence.ca website, and the Cochrane Database of Systematic Reviews (CDSR). We searched for individual studies in the MEDLINE, EMBASE, SCIRUS, Web of Science, PubMed, and the LILACS (Latin American and Caribbean Computer Library Center) databases. The Google Web search engine (http://www.google.com) and Google Scholar (http://www.scholar.google.com) were used to locate articles that may not have been included in the above databases. Grey literature was searched using ‘OpenSIGLE’, ‘NTIS’, ‘Health Management Information’, ‘British National Bibliography for Report Literature’, Proquest Dissertations and Theses - Full Text, Dissertation Abstracts, CINHAL, and CyberTesis.

We used the following text words and Medical Subject Headings: (a) *multiple sclerosis* OR *demyelinating disease*, and (b) s*urgery; surgical intervention; operations; medical history; hospitalisation; tonsillectomy; anaesthetic; appendectomy; adenoidectomy; risk factor; etiologic factor; severe; minor; association; causation; case–control; cohort.* In addition, reference lists of all relevant articles were examined for further pertinent studies; and conference proceedings were sought from various web sites and organizations. Forward citation searches of included studies and relevant literature reviews were also done. Primary authors and experts in the field were contacted to identify additional published, unpublished, or 'in-progress' studies. The search was not limited by publication date, language, or publication status. All databases were last accessed in August 2012.

### Inclusion criteria

We planned to include a broad range of observational studies: cohort, case–control, and cross sectional designs. As there were few primary studies, we also planned to include retrospective studies utilizing secondary data from healthcare databases. To be eligible for inclusion, studies needed to include patients with physician diagnosed MS (preferably by using diagnostic criteria for a definite diagnosis of MS) and report original data. Studies were excluded if there was no control group.

The primary outcome of interest was the development of MS following a past history of tonsillectomy, appendectomy, adenoidectomy, or other surgery. If the authors did not specify the type of surgery, we included these studies and classified them as “other surgery”. Surgeries were defined according to their classification in each individual study. Further, due to the estimated mean latency period and the critical age at puberty, these categories and sub-categories were divided by age at the time of surgery: (1) age ≤ 20 years, and (2) > 20 years.

### Data collection and analysis

#### Selection of studies

One of the study investigators (CL) performed the initial search of all databases to identify potentially relevant citations. Where it was not possible to accept or reject the study, the full text of the citation was obtained for further evaluation. Following the screening of titles and abstracts, the full texts of potential articles were retrieved (and translated into English where required) and assessed independently by two of the study investigators (CL, JKS). If any differences in opinion occurred, they were resolved by consensus with a third reviewer.

#### Data extraction and management

Data were independently extracted by one unmasked reviewer (CL) using a standardized electronic data collection form and were then checked by a second reviewer (JKS) for accuracy. When raw data were not provided, the data were extracted from figures; where necessary, we attempted to seek additional information from first or corresponding authors of the included studies via electronic mail. The following information was obtained for each study (where possible): source, eligibility, methods, participant demographics, MS diagnostic information, confounding variables adjusted for, outcome exposures, and results.

#### Quality assessment: Risk of bias in included studies

After identification of articles meeting the inclusion criteria, two review authors (CL, JKS) independently assessed the methodological quality of studies according to the criteria of the Newcastle-Ottawa Quality Assessment Scale (NOS) as recommended by the Cochrane Collaboration for assessing the quality of non-randomized studies [13]. The NOS is based on a cumulative score in each of three broad categories: selection of study groups, comparability of their cases and controls, and their ascertainment of the outcome / exposure on cases and controls. If a study fulfils the criteria for an item, a score of 1 point is allocated, with the exception of comparability which can score up to 2 points, resulting in a maximum score of 9. Similar to other reviews, we considered studies that received a score of ≥ 6 on the NOS criteria to be of high quality. We specifically classified studies as high risk of bias (1–3 points), medium risk of bias (4–5 points), or low risk of bias (6–9 points). In the case of disagreement between reviewers, differences were to be resolved by discussion until consensus was achieved.

#### Dealing with missing data

As missing data (statistics) were evident in many of the included studies, we attempted to contact 13 separate investigators at least twice. Six authors replied, and two provided the requested data; therefore, available data were extracted from published reports, and missing data were imputed. For those studies reporting “no significance”, with no additional statistical data, we assumed an odds ratio (OR) of 1.0 and estimated the confidence intervals (CIs) based on the number of reported MS cases [14]. Sensitivity analyses were performed to check the effect of imputation.

#### Assessment of heterogeneity and reporting bias

Heterogeneity between studies was examined visually using the I^2^ statistic. Deeks and colleagues (for the Cochrane Collaboration) [15] suggest the following as a rough guide for interpreting the I^2^ statistic:

•0% to 40%: might not be important;

•30% to 60%: may represent moderate heterogeneity;

•50% to 90%: may represent substantial heterogeneity;

•75% to 100%: considerable heterogeneity.

Possible sources of heterogeneity were assessed by sensitivity analyses and described qualitatively in Table 1 (Characteristics of included studies).

**Table 1:** Characteristics of included studies

**Author (year) Country**	**MS (n)**	**Control group (n)**	**F : M cases**	**Mean age at MS onset (years)**	**LAT**	**NOS Score**	**MS Dx criterion**	**Timing of surgery**	**Confounder variables**	**Exposure 1: Tonsil OR**^**1**^	**Exposure 2: Append OR**^**1**^	**Exposure 3: Adenoid OR**^**1**^	**Exposure 4: Other OR**^**1**^
										**(95% CI)**	**(95% CI)**	**(95% CI)**	**(95% CI)**
**Alonso** (2011) Iran	394	394	3.70	24.9	32	6	M	Pre	Age, sex	0.92 (0.51-1.64)	1.07 (0.52-2.19)	--	--
**Alter** (1968) USA	36	72			45	4	N	Pre	Age, sex, age at onset	--	--	--	0.78 (0.33-1.81)
**Anderson** (1981) Denmark	92	276	1.42	42.4	56	8	A; after 1994, Mc	≤ 20 y	Age, sex, month of birth, years in school	0.73 (0.32-1.64)	0.81 (0.22-2.98)	1.08 (0.64-1.82)	--
**Antonovsky** (1965) Israel	241	964	1.10		31	5	N	≤ 15 y, > 15 y	Age, sex, age of onset, region of birth	--	--	--	NS^2^
**Bansil** (1997) India	56	147	1.67	28	24	3	P	Pre	Birth place, religion, residence	--	--	--	0.97 (0.49-1.91)
**Berr** (1989) France	63	63	2.70	30.8	42	5	P	Pre	Age, sex, residence	1.0 (0.38-2.60)	--	--	--
**Bobowick** (1978) USA	10	8		29.7	40	6	N	≤ 20 y	Age, sex	2.50 (0.37-16.89)	5.0 (0.21-120.4)	--	--
**Broadley** (2000) England	595	372	3.16	28	52	1	N	Pre	None stated	1.02 (0.77-1.34)	--	--	--
**Casetta** (1994) Italy	104	150	2.0	32.2	44	5	Mc	≤ 15 y, > 15 y	Age (> 3 y), sex, residence	1.04 (0.62-1.76)	--	--	--
**Cendrowski** (1969) Poland	300	300		31.9	52	3	N	≤ 15 y	None stated	0.74 (0.43-1.27)	--	--	--
**Currier** (1982) Canada & USA	40	40	1.0	30	49	5	A	Pre	Age, sex	--	--	--	2.22 (0.86-5.74)
**Currier** (1974) Ireland	60	60	1.4	26	53	3	A	≤ 20 y	Age, sex, social class, marital status	1.18 (0.53-2.61)	1.46 (0.54-3.93)	--	1.45 (0.62-3.41)
**de Gennaro** (2009) Italy & Serbia	104	150	2.06	28	44	3	M	≤ 15 y, > 15 y	Age, sex, residence	≤ 15 y: 2.50	< 15 y: 1.60	--	--
										(1.42-4.41)*	(0.91-2.79)		
										> 15 y:	> 15 y:		
										2.20 (1.30-3.73)*	1.92 (1.09-3.41)*		
**da Silva** (2009) Brazil	81	81	2.10		22	4	N	Pre	Age, sex, place of birth	--	--	--	1.82 (0.95-3.51)
**Dolan** (2003) USA	24	24	2.0	35.4	42	3	P	≤ 20 y	Age, sex	--	--	--	2.14 (0.63-7.33)
**Dokuchaeva** (2006) Russia	178	178	2.80		48		No data	≤ 15 y, > 15 y	Age, sex, ethnic origin	≤ 15 y: 1.61 (1.01-2.56)	--	--	--
										> 15 y:			
										1.0 (0.64-1.56)			
**Gronning** (1993) Norway	155	200	1.50	--	60	4	B	≤20 y	Age, sex, residence	1.90 (1.15-3.16)*	1.10 (0.53-2.28)	--	--
**Gusev** (1996) Russia	155	155	1.63	25.8	56	3	Mc	≤ 15 y, > 15 y	Age, sex, residence, and ethnicity	0.85 (0.38-1.89)	--	--	--
**Hopkins** (1991) USA	14	56	4.30	35.2	41	7	P	Pre	Age, sex, race	--	--	--	NS^2^
**Koch** (1974) USA	7	7	2.50	29.3	46	4	N	Pre	None stated	--	--	--	1.0 (0.12-8.31)
**Koch-Henriksen** (1989) Denmark	297	297	1.42	32	56	8	A	≤ 15 y, > 15 y	Age, sex	≤ 15 y:	≤ 15 y:	≤ 15 y:	--
										0.89 (0.52-1.53)	2.19 (0.93-5.16)	0.85 (0.54-1.34)	
										> 15 y:	> 15 y:		
										1.49 (0.72-3.08)	1.24 (0.70-2.21)		
**Kurtzke** (1997) Norway	23	127	1.55	30	62	5	Schumacher committee	Pre	Age, sex	--	--	--	1.19 (0.48-2.95)
**Lamoureux** (1976) Canada	23	23	1.09			5	N	Pre	Age, sex, place of origin	3.56 (1.05-12.05)	3.05 (0.78-11.96)	--	--
**Lauer** (1994) Germany	150	150	2.04	30.3	51	4	B	≤ 14 y	Age, sex, type of residence	--	NS^2^	--	0.53 (0.32-0.86)*
**Martinez-Sobrepera** (2001) Cuba	50	50	4.50	--	21	5	P	Pre	Age, gender, skin color	--	--	--	1.50 (0.68-3.29)
**Poskanzer** (1980) USA	77	77	2.04	31	42	4	N	Pre	None stated	NS^2^	--	--	NS^2^
**Poskanzer** (1965) Scotland	210	210	1.31	33.6	60	8	A	≤ 20 y	Age, sex, residence	1.69 (1.15-2.50)*	1.42 (0.86-2.35)	--	--
**Roshanisefat** (2011) Sweden	20,542	204,157	1.87	46.3	--	6	MS register	≤ 20 y, > 20 y	Age, sex, age at onset, region	--	≤ 20 y:	--	--
											1.11 (0.94-1.31)		
											> 20 y:		
											1.02 (0.93-1.11)		
**Westlund** (1953) Canada	112	123	1.43	30.3	49	5	N	Pre	Age, sex, age at onset	--	--	--	2.26 (1.24-4.15)*
**YosefiPour** (2002) Iran	149	100	1.19	--	32	4	N	Pre	Age, sex	--	--	--	3.93 (1.97-7.84)*
**Zaadstra** (2008) Netherlands	2,821	2,550	2.30	--	52	7	N	< 25 y	Age, sex, education, residence	1.25 (1.11-1.40)*	--	--	--
**Zilber** (1996) Israel	70	64	1.73	25.2	31	6	M	≤ 20 y, Pre	Age, sex	≤ 20 y:	--	< 10 y: 3.0 (0.77-11.62)	0.58 (0.29-1.17)
										1.06 (0.47-2.39)			
										Pre: 0.96 (0.44-2.11)			
**Zorzon** (2003) Italy	140	131	1.72	31.2	45	5	M	Pre	None stated	--	--	--	0.46 (0.28-0.76)*

^**1**^ Odds ratio for combined surgeries using a random effects model.

^**2**^ Reported as not statistically significant, no data provided.

* Indicates statistically significant results.

**A** = Allison & Miller criteria, **Append** = appendectomy, **Adenoid** = adenoidectomy, **B** = Bauer criteria, **CI** = confidence interval, **Dx** = diagnosis, **LAT** = latitude, **Mc** = McAlpine criteria, **M** = McDonald criteria, **MS** = multiple sclerosis, **NOS** = Newcastle-Ottawa Scale, **N** = neurologist diagnosed, **NS** = not significant, **OR** = odds ratio, **P** = Poser criteria, **Pre**: Premorbid, age not specified other than surgery occurring before MS diagnosis, **Tonsil** = tonsillectomy, **Y** = years.

Stratified meta-regression, based on sub-groups including 10 or more studies, was performed to further examine heterogeneity. *A priori*, we planned to explore the following population-level continuous variables (geographic latitude, female-to-male MS case ratio, mean age of MS onset), and study-level dichotomous variables (language [English vs. non-English]), publication type [published vs. unpublished], number of covariates adjusted for [≤ 2 vs. > 2], total sample size [≤ 100 vs. > 100], and sample size of MS cases [≤ 100 vs. > 100]. Odds ratios (β) and 95% CIs were calculated using the study level log OR and the standard error (SE) of the estimate by constructing univariate random effects (RE) meta-regression models in STATA 12 using the *megareg* command. A plot of ORs was done against NOS scores to determine if there was a linear relationship between the methodological quality of the studies and their results [16]. We planned to explore publication bias and other potential reporting biases, in those pooled comparisons with 10 or more studies, using funnel plots. We used the graphical approach described by Peters et al. for assessing dichotomous outcomes with effects measured as odds ratios [17].

#### Effect measurement and data synthesis

Meta-analyses were performed using the Cochrane Collaboration software program Review Manager (Rev Man) Version 5.1 [18]. To estimate the strength of association between surgery exposure variables and risk for MS diagnosis, data were pooled using the inverse variance (IV) approach to calculate the OR and 95% CIs and statistical significance was set at *p* < 0.05. When interpreting results of the forest plots for dichotomous data, the area to the right side of the forest plot graph (> 1) favoured the control group. Studies were weighted based on sample size and the number of events.

Meta-analyses methods were selected based on study heterogeneity and the number of studies included in the analyses. When the I^2^ statistic was greater than 75%, we considered it substantial heterogeneity and pooled the study results using a RE model [19].

#### Subgroup analysis and investigation of heterogeneity

*A priori*, we planned to explore and address possible clinical heterogeneity as well as to investigate the magnitude and precision of effects by performing sub-group analyses based on type of surgery (tonsillectomy, appendectomy, adenoidectomy, other surgery), and age at time of surgery (≤ 20 years of age and those > 20 years of age). Sub-group analyses were also performed on reported association (number of covariates), female to male sex ratio, NOS score, latitude, and age at MS onset.

#### Sensitivity analysis

We performed sensitivity analyses by examining the results of the meta-analysis under different assumptions and checked for robustness of the observed findings. *A priori*, the following sensitivity analyses were planned:

1. By limiting included studies in the analyses to those with the highest methodological quality, do the results change?

2. For studies in which the OR was reported as “not significant” and therefore had to be imputed, do the results of the pooled analysis change if these are excluded from the results?

## Results

### Characteristics of included studies

**Figure 1:**
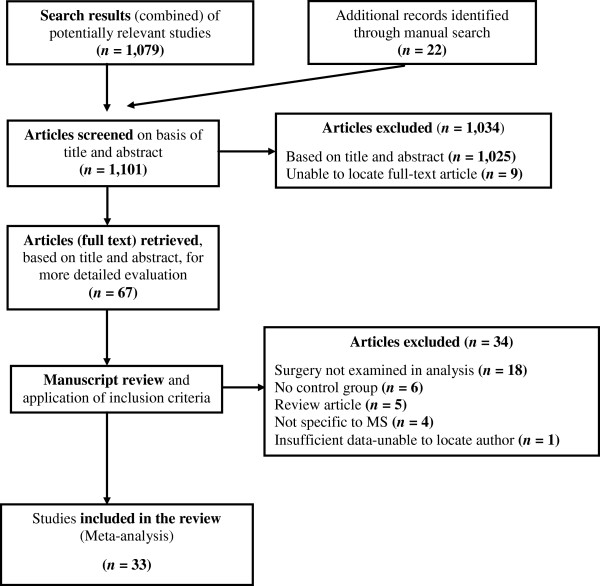
Flow diagram of study selection.

After excluding duplicate studies, we identified 100 individual studies, of which 75 were potentially relevant. Of these, despite our best attempts to contact primary or corresponding authors, eight studies could not be located [20-27]; therefore, 67 potentially relevant full text articles were retrieved for closer examination. Of the retrieved articles, 34 were excluded for the following reasons: 18 did not examine surgery as a risk factor [28-45]; six did not have a control group [46-51]; five were review articles [52-56]; four were not specific to MS [57-60]; and one had insufficient data, and we were unable to locate study authors [61]. A total of 33 studies were identified which met the inclusion criteria for the systematic review [62-94]. One additional cohort study was found but was not included due to heterogeneity in study design and exposure [95]. Figure 1 outlines the study selection process.

All of the included studies were case–control studies and were published between 1953 and 2011. Seventeen individual studies reported tonsillectomy data [62,64,67-69,71,73,75,76,78],[79,82,84,87,88,92,93], 11 reported appendectomy data [62,64,68,73,75,78,82,84],[85,87,89], three reported adenoidectomy data [64,82,93], and 18 reported on other surgeries [63,65,66,70,72-74,77,80,81],[83,85,86,88,90,91,93,94]. The majority of the studies (17 studies) were published in European countries [64,67,69-71,73,75,78,82,83],[85,87,89,92-94], followed by nine in North America [63,68,72,77,80,81,84,88],[90], four published in the Middle East [62,65,91,93], and lastly one each from India [66], Brazil [74], and Cuba [86].

Exposure risks for MS were reported as ORs in most of the studies while others simply reported whether the exposure risk for MS was “significant” or “not significant” [65,80,81,85,88]. The ORs ranged from 0.46 to 5.0 depending on the type of surgery reported. Eight independent studies reported statistically significant results for specific exposures [75,78,85,87,90-92,94], while the remaining 25 did not. See characteristics of included studies in Table 1.

### Quality assessment

Included studies were classified according to the NOS with seven studies considered high risk of bias [66,69,71,73,75,77,79]; 16 studies had a medium risk of bias [63,65,67,70,72,74,78,81],[83-86,88,90,91,94]; and nine studies had a low risk of bias [62,64,68,80,82,87,89,92],[93]. One study could not be assessed due to lack of included data [76].

### Exposure results

The meta-analysis included data from all 33 studies with 27,373 MS cases and 211,756 controls; there were twice as many females than males included in the studies and the mean age of MS diagnosis was 31 years. See Table 2 for pooled surgery exposure results.

**Table 2:** Odds ratios for pooled surgery exposures using a random effects model

**Exposure**	**Included studies (n)**	**MS case group (n)**	**Control group (n)**	**Total (n)**	**Level of heterogeneity I**^**2 **^**(χ**^**2 **^***p*)**	**Pooled OR (95% CI)**	**Test for overall effect**
							**Z ( *p *)**
Tonsillectomy ≤ 20 years	12	4,414	4,422	8,836	44**%** (0.05)	1.32 (1.08-1.61)^*^	2.76 (0.006) ^*^
Tonsillectomy > 20 years	9	1,801	1,618	3,419	32**%** (0.16)	1.20 (0.94-1.53)	1.45 (0.15)
Appendectomy ≤ 20 years	7	21,218	205,124	226,342	0**%** (0.54)	1.16 (1.01-1.34)^*^	2.06 (0.04) ^*^
Appendectomy > 20 years	5	21,360	205,021	226,381	46**%** (0.11)	1.26 (0.92-1.72)	1.43 (0.15)
Adenoidectomy ≤ 20 years	3	458	636	1,094	35**%** (0.21)	1.06 (0.68-1.68)	0.27 (0.79)
Surgery ≤ 20 years	4	485	1,202	1,687	19**%** (0.30)	0.95 (0.70-1.31)	0.29 (0.77)
Surgery > 20 years	15	1,099	1,371	2,470	71**%** (0.0001)	1.19 (0.83-1.70)	0.94 (0.35)

^*^ Indicates statistically significant results at *p* < 0.05.

**CI** = confidence interval**, MS** = multiple sclerosis, **OR** = odds ratio**.**

#### Tonsillectomy

Of the 17 separate studies reporting on tonsillectomy, 12 examined tonsillectomy at age ≤ 20 and 9 examined tonsillectomy at age > 20, or simply “premorbid”. Pooled analyses displayed statistical heterogeneity hence the estimates were based on the RE model.

**Figure 2:**
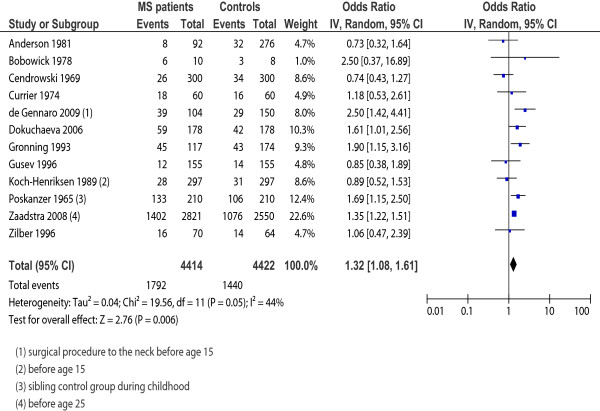
**Forest plot for tonsillectomy exposure occurring at ≤ 20 years of age.** Forest plot of 12 included studies reporting tonsillectomy in those ≤ 20 years of age in cases with multiple sclerosis and controls. *Horizontal lines*, 95% CIs of each study; *squares*, odds ratios of each individual study (the size represents the weight that the study was given in the meta-analysis); *diamond*, the pooled summary estimate; *solid vertical line*, null value. OR > one favoured the control group.

The 12 studies examining tonsillectomy at age ≤ 20 included 4,414 MS cases and 4,422 controls. The pooled RE model revealed moderate heterogeneity in the sample (I^2^ = 44%; *p* = 0.05) and a statistically significant relationship between tonsillectomy and MS onset (OR = 1.32; 95% CI, 1.08-1.61; *p* = 0.006). To further explore heterogeneity, we conducted a sensitivity analysis based on study quality (removing those studies with a NOS score < 6). Although removing the six studies with a NOS score of < 6 provided a more homogenous sample (I^2^ = 12%; *p* = 0.34) the difference was not significant as the direction and magnitude of the effect did not change (OR = 1.29; 95% CI, 1.08-1.53; *p* = 0.005); therefore, we included all 12 studies in the final analysis. Sensitivity analysis based on removing the one study where the OR was imputed did not change the significance nor magnitude of the effect (OR = 1.27; 95% CI, 1.02-1.58; *p* = 0.03); therefore, we included all 12 studies in the final analysis. Forest plot results for tonsillectomy exposure occurring at ≤ 20 years of age are presented in Figure 2.

The nine studies examining tonsillectomy in patients’ > 20 years or “premorbid” included 1,801 MS cases and 1,618 controls. The RE model revealed a homogeneous sample (I^2^ = 32%; *p* = 0.16) with no statistical difference between the groups (OR = 1.20; 95% CI, 0.94-1.53; *p* = 0.15). When four studies with a NOS score of < 6 were excluded, the pooled results became more homogeneous (I^2^ = 0%; *p* = 0.88) but the magnitude and the significance of the results did not change (OR = 1.04; 95% CI, 0.78-1.37; *p* = 0.85). All nine studies reported exact OR data thus, sensitivity analysis, based on the imputation of data, was not required and all nine studies were included in the final analysis.

#### Appendectomy

Eleven separate studies examined appendectomy; nine examined appendectomy in those ≤ 20 years and five examined appendectomy at age > 20, or “premorbid”. Pooled analyses displayed statistical heterogeneity, for that reason the estimates were based on the RE model.

The nine studies examining appendectomy in those ≤ 20 years included 21,574 MS cases and 205,480 controls. Moderate heterogeneity (I^2^ = 48%; *p* = 0.05) of the RE pooled results was demonstrated with no statistical relationship between appendectomy in those ≤ 20 and MS onset (OR = 1.16; 95% CI, 0.87-1.53; *p* = 0.31).We further conducted sensitivity analysis based on study quality. Although removing the five studies with a NOS score of < 6 provided a more homogenous sample (I^2^ = 13%; *p* = 0.33) the difference was not significant as the magnitude and precision of the effect did not change (OR = 1.21; 95% CI, 0.87-1.69; *p* = 0.27). Sensitivity analysis based on removing two studies where the ORs were imputed reduced the heterogeneity (I^2^ = 0%; *p* = 0.54) and changed the significance of the result (OR = 1.16; 95% CI, 1.01-1.34; *p* = 0.04) thus, we used the reduced, statistically significant model in the final analysis.

The five studies examining appendectomy occurring in those patients > 20 years or “premorbid” included 21,360 MS cases and 205,021 controls. The RE model revealed a moderately homogeneous sample (I^2^ = 46%; *p* = 0.11) with no statistical difference between groups (OR = 1.26; 95% CI, 0.92-1.72; *p* = 0.15). When studies with a NOS score of < 6 were excluded, the results did not change. All five studies reported exact OR data, consequently, sensitivity analysis based on the imputation of ORs was not required and all five studies were included in the final analysis.

#### Adenoidectomy

Three studies, involving 458 MS cases and 636 controls, reported on adenoidectomy in patient’s ≤ 20 years. The RE model revealed a homogeneous sample (I^2^ = 35%; *p* = 0.21) with no statistical difference between groups (OR = 1.06; 95% CI, 0.68-1.68; *p* = 0.79). All three studies were of high quality and all reported exact OR data, as a result, sensitivity analyses were not required.

#### Surgery

Eighteen discrete studies examined “other surgery”; four examined surgery occurring in those ≤ 20 years and 15 examined surgery in patients > 20, or “premorbid”. Pooled analyses displayed statistical heterogeneity thus the estimates were based on the RE model.

The four studies reporting on other surgery occurring in subject’s ≤ 20 years included 485 MS cases and 1,202 controls. The RE model revealed a homogeneous sample (I^2^ = 19%; *p* = 0.30) with no statistical difference between groups (OR = 0.95; 95% CI, 0.70-1.31; *p* = 0.77). Sensitivity analysis, based on study quality, was not done as all but one of the four studies had a NOS score of < 6. Further sensitivity analysis, excluding those studies that did not report a specific OR, did not significantly change the results (OR = 1.01; 95% CI, 0.28-3.57; *p* = 0.99).

The 15 studies examining other surgery in patients > 20, or “premorbid” included 1,099 MS cases and 1,371 controls. Significant heterogeneity (I^2^ = 71%; *p* < 0.0001) of the RE pooled results was demonstrated; regardless, there were no statistical differences between groups (OR = 1.19; 95% CI, 0.83-1.70; *p* = 0.35). Sensitivity analysis, based on study quality, was not done as all but two of the 15 included studies had a NOS score of < 6. Further sensitivity analysis, excluding those studies that did not report a specific OR, did not significantly change the results (OR = 1.32; 95% CI, 0.92-1.91; *p* = 0.14).

### Examining bias

To visually assess for heterogeneity, we plotted the ORs of high quality versus low quality studies. The plot showed no distinct linear relationship between methodological quality of studies (NOS score) and ORs, with no obvious clustering, indicating a low risk of bias.

For the tonsillectomy exposure (≤ 20 years), the test for publication bias was not statistically significant (*p* = 0.94), with a symmetrical funnel plot indicating a low risk for publication bias [96]. For the surgery premorbid exposure, the test for publication bias was also not statistically significant (*p* = 0.62); although, the plot was more asymmetrical indicating the potential for publication bias. Given the small number of studies included in the other exposures (< 10 studies), the interpretation of funnel plots must be undertaken with caution and are therefore not included here.

#### Meta-regression

Results from the meta- regression are presented in Table 3. Meta-regression was performed on the subgroups including at least 10 studies, namely tonsillectomy at age ≤ 20 and premorbid “surgery”. None of the variables entered into the regression analysis, for the surgery premorbid exposure, reached statistical significance therefore not significantly influencing the effect sizes. For the tonsillectomy at age ≤ 20, only one of the variables, the number of covariates adjusted for (≤ 2 vs. > 2), reached statistical significance (β = 1.64; 95% CI, 1.02-2.62). That is, the effect size or risk for MS diagnosis increased as the number of covariates that were adjusted for (in the individual included studies) increased. This further indicates limited residual heterogeneity after controlling for the influence of latitude, female to male sex ratio, mean age of MS onset, NOS score, language, publication status, and sample size. Although not a statistically significant finding (*p* = 0.45), the tonsillectomy exposure, in those ≤ 20 years, increased as the female to male ratio increased.

**Table 3:** **Sensitivity analyses and stratified meta-regression for assessing heterogeneity among case–control studies looking at the exposures tonsillectomy in childhood and “surgery” premorbid **^**1**^

**Exposure variables**	**Tonsillectomy - childhood**					**Surgery - premorbid**				
	**Studies**		**Sub-group**	**Meta-regression**		**Studies**		**Sub-group**	**Meta-regression**	
	***n***	**Total ( *n *)**	**OR (95% CI) **^**2**^	***p***	**β (95% CI) **^**3**^	***n***	**Total ( *n *)**	**OR (95% CI) **^**2**^	***p***	**β (95% CI) **^**3**^
**Population-level characteristics**										
**Latitude**	12	8,836	1.32 (1.08-1.61)^*^	0.79	1.00 (0.96-1.03)	15	2,470	1.19 (0.83-1.70)	0.89	1.00 (0.97-1.03)
(geographic)										
**Female: Male**	10	8,218	1.39 (1.15-1.68)^*^	0.45	1.20 (0.71-2.04)	14	2,362	1.22 (0.84-1.79)	0.91	0.98 (0.64-1.48)
**Mean age of MS onset**	9	2,818	1.17 (0.85-1.61)	0.51	0.98 (0.90-1.06)	10	1,551	1.12 (0.77-1.63)	0.53	0.94 (0.77-1.16)
**Study-level characteristics**										
**NOS score**	11	8,480	1.29 (1.03-1.60)^*^	0.77	0.98 (0.85-1.13)	15	2,470	1.19 (0.83-1.70)	0.58	0.89 (0.58-1.37)
**NOS score** (< 6 vs. ≥ 6)	12	8,836	1.32 (1.08-1.61)^*^	0.60	0.88 (0.51-1.50)	15	2,470	1.19 (0.83-1.70)	0.84	0.84 (0.13-5.57)
**Language** (English vs. non-English)	12	8,836	1.32 (1.08-1.61)^*^	0.10	0.62 (0.34-1.12)	15	2,470	1.19 (0.83-1.70)	0.73	0.78 (0.17-3.61)
**Pub type** (published vs. unpublished)	12	8,836	1.32 (1.08-1.61)^*^	0.09	0.50 (0.22-1.13)	15	2,470	1.19 (0.83-1.70)	N/A^**4**^	
**Number of covariates **^**5**^ (≤ 2 vs. > 2)	12	8,836	1.32 (1.08-1.61)^*^	0.04	1.64 (1.02-2.62)^*^	15	2,470	1.19 (0.83-1.70)	0.73	0.88 (0.39-1.96)
**Sample size** (≤ 100 vs. > 100)	12	8,836	1.32 (1.08-1.61)^*^	0.54	0.52 (0.05-5.27)	15	2,470	1.19 (0.83-1.70)	0.56	0.76 (0.29-2.01)
**MS cases** (≤ 100 vs. > 100)	12	8,836	1.32 (1.08-1.61)^*^	0.37	1.32 (0.68-2.60)	15	2,470	1.19 (0.83-1.70)	0.70	0.87 (0.40-1.90)

^**1**^ All analyses weighted by sample size.

^**2**^ OR (95**%** CI) were calculated using the random effects model in RevMan.

^**3**^ β (95**%** CI) were calculated using the study level log OR and the SE of the estimate (calculated in RevMan) by univariate random effects meta-regression in STATA 12 using the *megareg* command.

^**4**^ All studies were published – analysis not warranted.

^**5**^ Number of potentially confounding variables adjusted for in individual studies.

**CI** = confidence interval**, MS** = multiple sclerosis**, NOS** = Newcastle-Ottawa Scale, **OR** = odds ratio, **Pub** = Publication, **SE** = Standard error.

## Discussion

Overall, the evidence presented in this review supports a relationship between tonsillectomy and appendectomy in childhood and the subsequent risk of MS. More specifically, those with tonsillectomy or appendectomy at ≤ 20 years of age were approximately 30% more likely to be diagnosed with MS in comparison to similar patients who did not have a tonsillectomy or appendectomy at ≤ 20 years of age. The findings of this review do not support an association between tonsillectomy or appendectomy occurring in those > 20 years, adenoidectomy, and “other surgery” occurring at any age and the subsequent risk for the onset of MS. Although we did not find an association between these variables and risk for MS, it is important to note that due to the multi-factorial and heterogeneous nature of MS, surgery may indeed pose a slight risk for certain individuals.

Several theories linking tonsillectomy to MS risk have been postulated over the years. Meurman and Wising [39] proposed tonsillectecomy as a possible risk factor for MS in that an upper respiratory tract infection may trigger MS or that a locally deficient immune system may facilitate the invasion of an etiologically relevant, infectious agents. Tonsillectomy may leave sufficient lymphoid tissue adjacent to the central nervous system to instigate the exaggerated immune response seen in MS [39,69,87]. Poskanzer (1965) was particulary interested in examining tonsillectomy and MS risk at it predisposes to the development of neurological complications in poliomyelitis, another infection that is much more widespread than is suggested by its neurological manifestations [87,97-99].

Of particular interest is the association between recurrent tonsillitis and EBV infection and reactivation [100]. A recent meta-analysis of case–control and cohort studies found a statistically significant combined relative risk (RR) for MS in those with a past history of infectious mononucleosis (RR = 2.17; 95% CI, 1.97-2.39) [101]. Furthermore, others have found statistically significant elevated levels of antibody to some common infectious agents, other than EBV, in children and adults with MS compared to controls [92,102-104]. Krone and collegues [104] postulate that these findings reflect a dysregulation of immune function as a consequence of the development of the disease. They assert that immune dysregulation in MS is likely to be an early event preceding the onset of MS disease by many years or even decades [14,105,106]. It is likely that the elevated antibody concentrations do not directly cause MS but rather reflect a shift in patterns of immune reactivity away from protection towards enhancement of the risk of disease. These authors suggest that studies on MS-associated infectious agents could lead to the identification of specific antigenic determinants involved in the generation and maintenance of immune dysregulation.

There seems to be similarities between MS and appendicitis, which often results in appendectomy. More recently, scientists have recognized the role of the appendix as part of the body’s immune system, as it contains white blood cells and acts as a reservoir for “good” bacteria for the gut [107]. The role of the appendix is to contain “good bacteria”; when bacteria in the intestines die or are purged from dysentery or cholera, the “good bacteria” are replaced from the stores in the appendix. As a result, appendicitis (inflammation of the appendix) may indicate inadequate immunological function [84,89,108]; thereby providing an association of both EBV and appendicitis (resulting in appendectomy) with MS [109-111]. Immunological reaction relevant to appendicitis may indicate MS risk as activation of peripheral blood mononuclear cells, including those causing inflammatory destruction of myelin, which occurs in lymphoid tissue [89,108]. Appendicitis is a marker of immune characteristics influencing immune-mediated disease risk, as it has a direct role in the development of ulcerative colitis [89,112,113]. Further, CD8+ T-cell deficiency is a feature of both ulcerative colitis and MS [114].

There seems to be other possible similarities between MS and appendicitis. First, appendicitis and MS are autoimmune diseases. Both appendicitis and MS are diseases associated with industrialized countries but not developing countries [115,116]. MS is a disease largely prevalent in Europe, North America, Australia, and New Zealand, but is rare among Asians and Africans [116]. Lastly, several epidemiological and experimental studies support the hygiene hypothesis in both appendicitis [115,117] and MS [109,118], which postulates that immunopathology may be an unanticipated consequence of advances in sanitation and public health [109,118,119].

### Quality of the evidence

One of the main challenges of the review related to the varying definitions of “surgery” in the included studies. Some studies included only tonsillectomy or appendectomy, and some included all surgeries. More importantly, the number of surgeries in individual patients (prior to MS onset) would have been a significant variable to assess; however, none of the included studies reported this as a potential risk factor. Another potential limitation pertained to the fact that, with the exception of a single study [89], the majority of studies reporting on appendectomy did not indicate the reason for surgery; therefore, we had to assume that appendectomy was as a result of appendicitis (infection / inflammation). It must be emphasized that characterizing appendectomy, by underlying diagnosis, may be important when assessing true MS risk.

Ideally, in case–control studies, cases should be representative of the entire population and be identified by independent MS diagnostic criteria. Controls should be randomly selected within the community of the residing representative MS cases. Only nine case–control studies examined in this review selected controls from the community [64,65,67,79,80,82,88,90],[93]. To further control for bias, control subjects should have no prior history of disease, representing healthy individuals of the same socio-economic class.

Age at disease onset is very important in MS etiologic studies and ought to have been used as a matching variable to potentially control for bias. The age at disease diagnosis is used to allow researchers a timeframe to assess for potential exposure to associated agents. Ideally, case–control matching ought to be done for universal variables such as age, sex, and age at MS diagnosis. Other matching variables to be considered in MS studies include residence and place of birth. Interviewer blinding, another means of controlling for bias, was done by only one study [94].

### Potential bias in the review process

The two review authors who assessed the methodological quality were not blinded for authors, journal, or institution. The potential bias caused by the non-blinded quality assessment was expected to be low as neither review author had a conflict of interest. Specifically, the review authors did not have any (financial or other) interest in positive or negative results. Furthermore, we searched the grey literature extensively for eligible studies, presented the search strategy and the inclusion criteria list, and all of the final results of the assessment, so that readers can make their own determinations of the results and our conclusions.

There is a possibility of publication bias or study selection bias in this meta-analysis. For example, by missing unpublished negative studies we may be over-estimating the association between prior surgery and the risk for developing MS. However, a comprehensive search of the published literature for potentially relevant studies was conducted, using a systematic strategy to avoid bias. This was followed by attempts to contact corresponding and first authors, as we recognize that unpublished or negative studies may exist.

## Conclusion

This result of this meta-analysis suggests a statistically significant and clinically important increased risk for developing MS in those with tonsillectomy and appendectomy at ≤ 20 years of age. Despite this significant finding, this in no way suggests or demonstrates causality, in that epidemiological studies can only provide etiological clues at best. More rigorous prospective studies, with high statistical power, are needed to prove an effect. Future prospective studies, that take into consideration the long latency period between the age of putative biological onset and clinical onset of MS, are needed in order to definitively rule out any links between tonsillectomy (or other surgeries) and MS.

## Competing interests

The authors have no financial, personal, or any other kind of competing interests with this paper.

## Authors’ contributions

Study concept and design: CL, JAK-S, and SF; acquisition and preparation of data: CL; analysis and interpretation of the data: CL, JAK-S, and SF; risk of bias assessment: CL and JAK-S; first draft of the manuscript: CL. All authors critically reviewed the manuscript and approved the final version of the manuscript to be published.

## Pre-publication history

The pre-publication history for this paper can be accessed here:

http://www.biomedcentral.com/1471-2377/13/41/prepub

## Authors’ note

We would be grateful if readers would send us reprints of any published or unpublished studies on multiple sclerosis and surgeries that have not been included in this paper, as well as any additional data missed from the included studies.
